# Translational Pharmacokinetic/Pharmacodynamic Model for mRNA-3927, an Investigational Therapeutic for the Treatment of Propionic Acidemia

**DOI:** 10.1089/nat.2022.0036

**Published:** 2023-03-30

**Authors:** Husain Attarwala, Matthew Lumley, Min Liang, Vijay Ivaturi, Joe Senn

**Affiliations:** ^1^ Moderna, Inc., Cambridge, Massachusetts, USA.; ^2^ Pumas AI, Inc., Centreville, Virginia, USA.

**Keywords:** propionic acidemia, propionyl-CoA carboxylase, mRNA therapy, lipid nanoparticles

## Abstract

Propionic acidemia (PA) is an ultrarare disorder caused by deficiency of the mitochondrial enzyme, propionyl-CoA carboxylase (PCC), composed of PCCA and PCCB subunits. An enzyme replacement therapy is being developed using dual messenger RNA (mRNA) therapy composed of lipid nanoparticles (LNPs) encapsulating mRNAs encoding PCCA and PCCB subunits of the PCC enzyme. We herein report on development of a translational semimechanistic pharmacokinetic (PK) and PK/pharmacodynamic (PD) model to quantify the relationship between the mRNA components of mRNA-3927 (an LNP encapsulating PCCA and PCCB mRNAs) and dose levels; PCCA/B mRNA PK and PD responses were assessed as circulating levels of primary disease markers 2-methyl citrate, 3-hydroxypropionate, and propionyl carnitine normalized to acetyl carnitine (C3/C2 ratio) to inform the first-in-human dose range and regimen selection. The translational PK/PD model was developed using preclinical data available in mice with PA, Sprague Dawley rats, and cynomolgus monkeys at dose levels ranging from 0.2 to 9 mg/kg. PCCA/B mRNA PK in mice, rats, and monkeys was adequately described using allometric scaling of volume and clearance parameters. The interspecies preclinical model was scaled allometrically to humans to predict the dose–response relationship in adult and pediatric patients with PA to guide selection of dose range and regimen for the Phase 1 clinical trial (ClinicalTrials.gov Identifier NCT04159103).

## Introduction

Propionic acidemia (PA) is an ultrarare metabolic disorder that manifests during infancy and early childhood and is characterized by a deficiency of propionyl-CoA carboxylase (PCC), an enzyme that catalyzes the conversion of propionyl-CoA to methylmalonyl-CoA. The pathophysiology of PA stems from defective or deficient expression of PCC protein, which is encoded by *PCCA* and *PCCB* genes. Dysfunction in the PCC enzyme results in a metabolic block in the propionate metabolism pathway, leading to metabolic acidosis marked by elevated levels of metabolites, including 2-methyl citrate (2-MC), 3-hydroxypropionate (3-HP), and propionyl carnitine normalized to acetyl carnitine (C3/C2) ratio [1,2]. There are two disease subtypes classified based on deficiency of either the PCCA subunit (Type I) or the PCCB subunit (Type II): the dual messenger RNA (mRNA) targets both disease subtypes [2–6].

Previously published data using a disease model of PA in mice provided compelling preclinical proof of concept for dual mRNA therapy (mRNA-3927) as a potential disease-modifying treatment that addresses the underlying metabolic defect for PCCA- and PCCB-deficient subtypes of PA. These studies also comprehensively aided in establishing the long-term efficacy and safety of a systemically administered combination mRNA therapeutic as an enzyme-replacement approach. The data described therein support the clinical development of mRNA-3927 as a therapeutic for this devastating pediatric disorder [7].

Herein, we report on the development of a translational semimechanistic pharmacokinetic (PK)/pharmacodynamic (PD) model of mRNA-3927. The purpose of the integrated analyses was to quantitatively characterize the PK and PD responses of mRNA-3927 in nonclinical species and predict the dose–response relationship between mRNA-3927 and PD endpoints in adult, adolescent, and pediatric patients with PA to guide dose selection for the Phase 1 clinical trial.

## Materials and Methods

### Data used for model development

#### Pharmacokinetic studies

PCCA mRNA data were available from mice deficient in PCC [(Strain Code: 4011020 (A138T) PCCA −/−); *N* = 111], juvenile Sprague Dawley rats (*N* = 19), and cynomolgus monkeys (*N* = 16). Mice received a single intravenous (IV) bolus dose of 1 and 2 mg/kg of mRNA-3927. Juvenile Sprague Dawley rats received 3 IV bolus doses at 1, 3, and 9 mg/kg every 2 weeks. Cynomolgus monkeys received 3 IV bolus doses at 1, 3, and 5 mg/kg every 2 weeks. Blood samples were collected at predetermined time points postdose: up to 48 h from mice and up to 96 h from juvenile Sprague Dawley rats and cynomolgus monkeys. Experimental protocols were approved by the Institutional Animal Care and Use Committee at Moderna, Inc., and complied with all relevant ethical regulations regarding the use of research animals.

#### Pharmacodynamic studies

The PD of mRNA-3927 was evaluated in mice deficient in PCC [(Strain Code: 4011020 (A138T) PCCA −/−); *N* = 111] after a single IV dose or multiple IV doses. The PD response of mRNA-3927 was assessed as hepatic production of PCC protein and plasma concentrations of 2-MC, 3-HP, and C3/C2 ratio levels after a single IV bolus dose of mRNA-3927 (0.2, 0.5, 1, and 2 mg/kg) or after multiple IV bolus doses of mRNA-3927 (0.5 and 2 mg/kg) administered once every 3 weeks (q3W) for 12 weeks (total of 4 doses).

### Model development

**FIG. 1.**
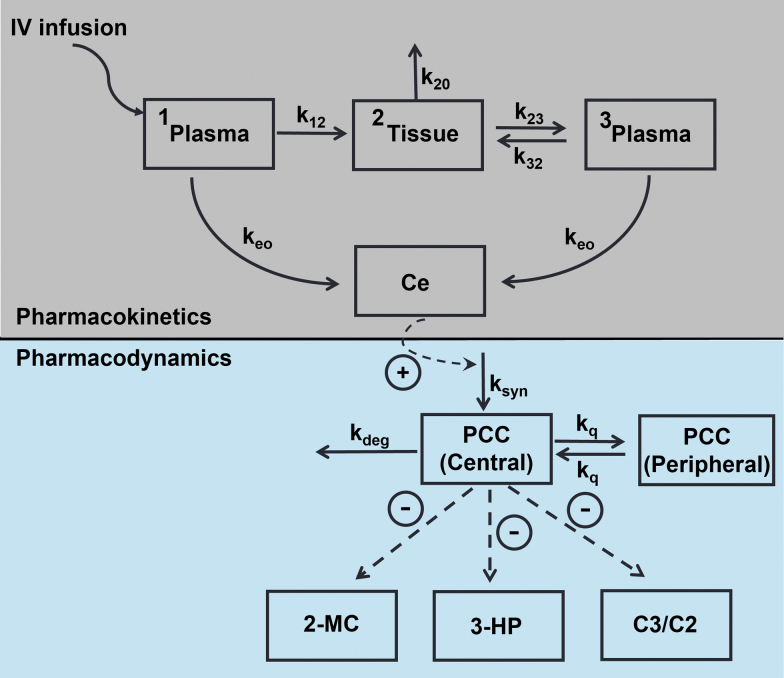
PK/PD model schematic. k_12_, transfer rate from plasma compartment 1 to tissue compartment; k_20_, rate of elimination from tissue compartment; k_23_, transfer rate from tissue compartment to plasma compartment 2; k_32_, transfer rate from plasma compartment 2 to tissue compartment; Ce, effect compartment concentration of mRNA-3927; k_deg_, PCC protein degradation rate; k_e0_, equilibrium rate constant for effect compartment; k_q_, intercompartmental rate constant for PCC protein; k_syn_, PCC protein synthesis rate; PCC, propionyl-CoA carboxylase.

The overall model development was executed in a stepwise manner (Fig. 1) as follows: (1) Plasma concentration–time profiles of PCCA/B mRNA in mice, rats, and monkeys were modeled using a semimechanistic PK model with allometric scaling of volume and clearance parameters; (2) PCC protein expression rate was modeled as a linear function of plasma PCCA/B mRNA concentration using a 2-compartment indirect response PD model with an empirical effect compartment; (3) PCC protein-mediated reduction of 2-MC, 3-HP, and C3/C2 ratios was modeled using a direct sigmoidal maximum inhibition (*I*_max_) model; and (4) the PK/PD model was extrapolated to humans using allometric scaling of PK parameters, and the expected plasma 2-MC profiles after q3W dosing at various dose levels were simulated to guide selection of doses for the first-in-human clinical study. Model equations are provided in Supplementary Table S1.

Population analyses were conducted using nonlinear mixed-effects modeling using Pumas software, Version 2.0 (Pumas AI). The first-order conditional estimation with interaction (FOCEI) method was employed for all model runs, except for the 3-HP PD model, where the Laplacian method was used. Data sets were prepared using R 3.6. Graphics were constructed through either R or the program GraphPad Prism 8.2. Assessments of model adequacy were guided by goodness-of-fit criteria, including (1) visual inspection of diagnostic scatterplots (observed vs. predicted concentration, residual/weighted residual vs. predicted concentration or time), (2) successful convergence of the minimization routine, (3) plausibility of parameter estimates, and (4) visual predictive checks.

## Results

### Pharmacokinetic model for PCC mRNA

**FIG. 2.**
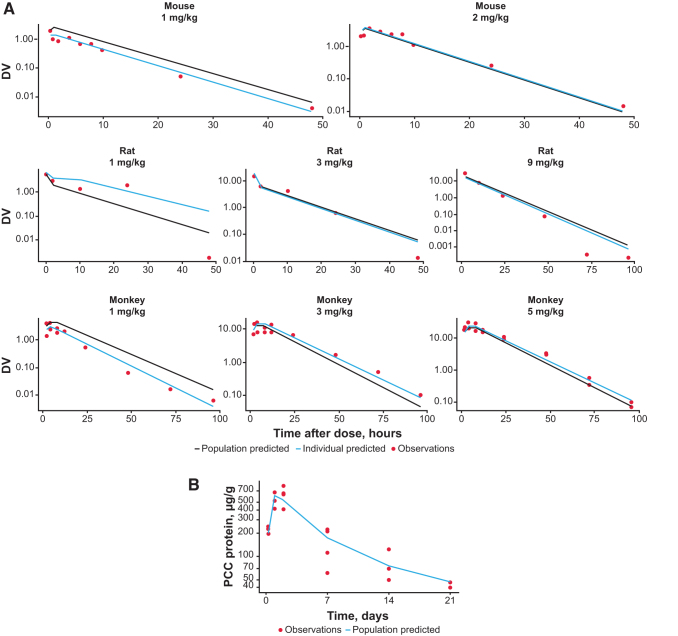
Observed and fitted **(A)** concentration–time profiles of mRNA-3927 in mice, rats, and monkeys, and **(B)** PCC protein–time profile after 1 mg/kg IV bolus dose of hPCCA+hPCCB mRNA in PCC-deficient mice. *Black lines* indicate population predicted, *blue lines* indicate individual predicted, and *closed circles* indicate observations. DV, dependent variable (plasma concentration); IV, intravenous; mRNA, messenger RNA; PCC, propionyl-CoA carboxylase.

**FIG. 3.**
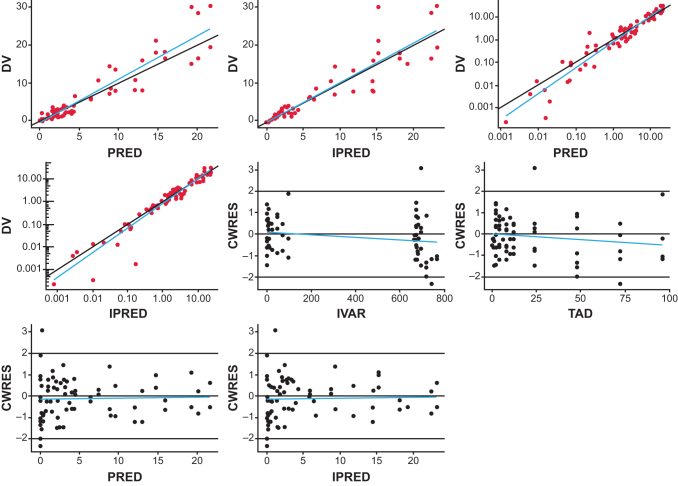
PK model goodness-of-fit plots. *Blue lines* indicate linear fit. CWRES, conditional weighted residuals; DV, dependent variable (plasma concentration); IPRED, individual predicted; IVAR, independent variable (time); PK, pharmacokinetic; PRED, population predicted; TAD, time after dose.

A semimechanistic PK model was found to suitably describe the observed delayed *C*_max_ in nonhuman primates where maximal PCC mRNA (PCCA+PCCB mRNA) concentrations were reached 4 h after mRNA-3927 IV infusion (data not shown), suggesting a distribution and redistribution PK pattern. Plasma concentration–time profiles of PCC mRNA from mice, rats, and monkeys were fitted simultaneously to the semimechanistic PK model to estimate all model parameters, including body weight–based allometric exponents for clearance. The population PK model converged successfully with reasonable percentage of residual standard error (% RSE) estimates. Parameter estimates are shown in Table 1. Model fits and goodness-of-fit plots are shown in Figs. 2A and 3, respectively. Model diagnostics suggested that the model adequately described the PCCA mRNA PK in mice, rats, and monkeys with a lack of any systematic bias.

**Table 1.** Model Parameter Estimates

Parameter (unit)	Model estimate (% RSE)	
PK model parameter estimates		
tvCL_12_ (mL/h)^a^	19.7 (23.1)	
tvCL_23_ (mL/h)^b^	0.215 (35.9)	
tvCL_32_ (mL/h)^c^	2.96 (48.2)	
tvCL_20_ (mL/h)^d^	0.136 (3.78)	
tvV (mL)^e^	2.67 (Fixed)^f^	
tvV_2_ (mL)^g^	0.961 (Fixed)^f^	
Clα^h^	0.631 (9.2)	
Clβ^i^	1.10 (7.55)	
IIV on CL_32_ (%)	52.7	
Proportional residual error (%)	37.5 (11.0)	

Parameter (unit)	Model estimate (% RSE)	
mRNA-3927 PCC PK/PD model parameter estimates		
k_e0_ (1/h)^j^	0.242 (3.35)	
Slope [(μg/g/h)/(μg/mL)]	55.6 (1.93)	
k_deg_ (1/h)^k^	0.0075 (2.82)	
k_q_ (1/h)^l^	0.00474 (6.7)	
Proportional residual error (%)	30.5 (0.02)	

Parameter (unit)	Model estimate (% RSE)	IIV (%)
Plasma 2-MC, 3-HP, and C3/C2 PD model parameter estimates		
2-MC^m^		
E0_2-MC_ (μM/L)^n^	1.67 (4.46)	
2**-**MC_base_ (μM/L)^o^	2.93 (6.66)	30.0
IC50_2**-**MC_ (μg/g)	21.0 (8.96)	45.4
Proportional residual error (%)	23.4 (3.84)	
3-HP^m^		
E0_3-HP_ (μM/L)^n^	0.0987 (6.74)	
3-HP_base_ (μM/L)^o^	60.1 (14.4)	51.1
IC50_3-HP_ (μg/g)	37.5 (12.2)	73.8
Proportional residual error (%)	34.8 (5.56)	
C3/C2 ratio^m^		
E0_C3/C2_ (μM/L)^n^	0.347 (6.4)	
C3/C2_base_ (μM/L)^o^	2.31 (6.61)	51.9
IC50_C3/C2_ (μg/g)	32.1 (11.7)	56.4
Proportional residual error (%)	36.0 (4.79)	

^a^ tvCL_12_, typical value for clearance from compartment 1 to compartment 2.

^b^ tvCL_23_, typical value for clearance from compartment 2 to compartment 3.

^c^ tvCL_32_, typical value for clearance from compartment 3 to compartment 2.

^d^ tvCL_20_, typical value for tissue elimination clearance.

^e^ tvV, typical value for distribution volume of compartment 1 and 3.

^f^ V and V_2_ were first estimated and then fixed in the subsequent step to allow % RSE computation.

^g^ tvV_2_, typical value for distribution volume of compartment 2.

^h^ Clα, allometric exponent for CL_12_ and CL_32_ parameters (plasma → tissue clearance parameters).

^i^ Clβ, allometric exponent for CL_23_ and CL_20_ parameters (tissue → plasma clearance parameters).

^j^ k_e0_, equilibrium rate constant for effect compartment.

^k^ k_deg_, PCC protein degradation rate.

^l^ k_q_, intercompartmental rate constant for PCC protein.

^m^ Maximum inhibition (*I*_max_) was fixed at 0.999.

^n^ Plasma biomarker levels not affected by mRNA-3927.

^o^ Baseline biomarker level amenable to suppression.

2-MC, 2-methyl citrate; 3-HP, 3-hydroxypropionate; C2, acetyl carnitine; C3, propionyl carnitine; IIV, interindividual variability; mRNA, messenger RNA; PCC, propionyl-CoA carboxylase; PD, pharmacodynamic; PK, pharmacokinetic; RSE, residual standard error; tv, typical value.

### PK/PD model for hepatic PCC protein

A liver concentration–time profile of PCC protein (PCCA+PCCB) was described using a 2-compartment indirect response PD model where the synthesis rate of PCC protein increased linearly with an increase in plasma PCC mRNA concentration through an effect compartment.

An effect compartment was incorporated into the semimechanistic model for population PK/PD to account for the slight hysteresis or delayed onset and longer duration of effect observed after IV administration of mRNA-3927 lipid nanoparticles (LNPs). Furthermore, a 2-compartment model for PCC protein was needed to adequately capture the biexponential decline of PCC protein observed in the terminal phase.

The PK/PD model converged successfully, and parameters were estimated with reasonable % RSE, indicating the reliability of the estimated parameters' value (Table 1). The observed and fitted PCC concentration–time profile is shown in Fig. 2B, which indicates that the model adequately described the PCC synthesis and time course in mice after an IV bolus dose. The model estimated a half-life of PCC protein of ∼7 days.

### PK/PD model for plasma 2-MC, 3-HP, and C3/C2 ratio in PCC-deficient mice

**FIG. 4.**
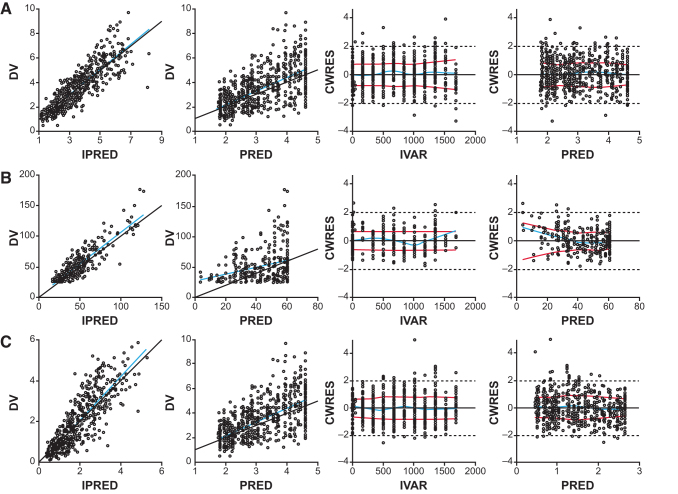
Goodness-of-fit plots for the plasma **(A)** 2-MC, **(B)** 3-HP, and **(C)** C3/C2 PD model. *Blue lines* indicate linear fit. 2-MC, 2-methyl citrate; 3-HP, 3-hydroxypropionate; C2, acetyl carnitine; C3, propionyl carnitine; CWRES, conditional weighted residuals; DV, dependent variable (plasma concentration); IPRED, individual predicted; IVAR, independent variable (time); PD, pharmacodynamic; PRED, population predicted.

The transitional semimechanistic PK/PD model converged successfully with reasonable % RSE estimates. Parameter estimates are shown in Table 1. Model fitted individual plots are shown in Supplementary Figs. S1–S3 and goodness-of-fit plots are shown in Fig. 4; these data indicate that the model adequately describes the plasma 2-MC, 3-HP, and C3/C2 responses in mice with a lack of systematic bias. Visual predictive check for 3-HP at doses of >0.5 mg/kg showed that the model-predicted median was greater than that for the observed data (data not shown); this result was likely due to limitations of the preclinical 3-HP assay because several observations were below the limit of quantification of 25 μM/L after administration of mRNA-3927.

The concentration of liver PCC protein needed for 50% inhibition of plasma 2-MC, 3-HP, and C3/C2 was estimated as 21.0, 37.5, and 32.1 μg/g, respectively. The 2-MC_base_, 3-HP_base_, and C3/C2_base_ parameters represent plasma levels of corresponding biomarkers that are susceptible to suppression by PCC protein expressed through mRNA-3927 in mice; the E0_2-MC_, E0_3-HP_, and E0_C3/C2_ parameters represent plasma levels of corresponding biomarkers that are not susceptible to PCC expressed after IV administration of mRNA-3927. Based on parameter estimates in Table 1, ∼63.7%, 99.8%, and 86.9% of total plasma 2-MC, 3-HP, and C3/C2, respectively, was amenable to suppression by mRNA-3927–mediated expression of PCC protein in PCC-deficient mice. Near maximal suppression of plasma 2-MC, 3-HP, and C3/C2 ratio was maintained at q3W doses of ≥2 mg/kg in mice. The variability (90% prediction interval) in biomarker suppression was predicted to reduce with an increase in dose.

### Extrapolation of the PK/PD model to humans

The preclinical PK/PD model described earlier was extrapolated to humans based on allometric scaling of model parameters. Based on the PCCA/B mRNA PK model described earlier, estimated allometric exponent was 0.63 for CL_12_ and CL_32_ and 1.1 for CL_23_ and CL_20_. Allometric exponent for the volume of distribution parameters (ie, V and V_2_) was fixed to 1.

## Discussion

Safe and effective therapies for the treatment of rare diseases remain a substantial unmet medical need. PA is an ultrarare devastating pediatric disorder caused by PCC enzyme deficiency that results in impaired propionate metabolism and the abnormal accumulation of toxic metabolites in the body [1,2]. mRNA-3927 is a novel enzyme replacement therapy consisting of dual mRNAs encoding a functional PCC enzyme [7]. mRNA technology has transformed the therapeutic landscape in terms of mechanism of drug delivery and action, utilizing systemic delivery of mRNA through LNPs and allowing the endogenous translational machinery of the cell to produce therapeutic proteins [8–10].

The aim of the current investigation was to develop a translational PK/PD model to guide dose selection for the first-in-human clinical study in patients with PA. We developed a translational semimechanistic PK and PK/PD model to quantify the relationship between mRNA-3927 dose, PCCA/B mRNA, and PK and PD responses to guide dose selection for the Phase 1 clinical study of mRNA-3927 in adult, adolescent, and pediatric patients with PA.

In our analyses, we took a stepwise approach to developing the translational model, initially characterizing the PK of PCCA/B mRNA in mice, rats, and monkeys. Our results demonstrated that the plasma PK of PCCA/B mRNA was adequately described by a semimechanistic PK model, and that allometric scaling of PK parameters adequately described the plasma concentrations of PCCA/B mRNA. Next, in the PD assessments of mRNA-3927 in mice deficient in PCC, PCC protein expression profiles were shown to be adequately described using a linear function of plasma PCCA/B mRNA concentration through an empirical effect compartment.

The model estimated a PCC protein half-life of 7 days. PCC protein-mediated reduction of 2-MC, 3-HP, and C3/C2 ratio was also adequately described by the model. Evaluation of relationship between mRNA-3927 dose and PD responses showed that near maximal suppression of plasma 2-MC, 3-HP, and C3/C2 ratio was maintained at q3W doses of 2 mg/kg in mice. To inform starting dose and dose range selection for the first-in-human clinical study in patients with PA (>1 year of age), this preclinical PK/PD model was subsequently extrapolated to humans based on allometric scaling of model parameters and was used to simulate PD responses at different dose levels and dosing intervals.

Although this model could successfully predict peak biomarker reductions at steady state and recovery in humans, further refinements were limited due to heterogeneity of residual PCC enzyme levels in animal models and by the sparse literature on the biochemical interplay between 2-MC, 3-HP, and C3/C2 containing pathways. Regardless of these limitations, the semimechanistic model suitably predicted observed biomarker responses regardless of the investigational species.

Toxic accumulation of 3-HP, methylcitric acid, and/or methylmalonic acid in plasma, urine, and other body fluids due to deficiency of PCC results in devastating clinical complications in patients with PA, affecting neurologic, cardiologic, hematologic, immunologic, and gastrointestinal systems [11,12]. Current management of patients with PA is inadequate and does not address the underlying defect of the disorder [13]. Carglumic acid, a structural analogue of *N*-acetylglutamate that activates the first enzyme in the urea cycle, is approved in the European Union for the treatment of hyperammonemia due to PA [14].

Recent preclinical studies comparing dual mRNAs (mRNA-3927) with carglumic acid showed that although plasma ammonia was normalized in mice treated with either agent, only dual mRNAs resulted in reductions in plasma 2-MC, 3-HP, and C3/C2 ratio, as well as reductions in tissue 2-MC and increased PCC activity in the liver [7]. Thus, unlike carglumic acid, dual mRNAs addressed the underlying defect in PA by restoring propionate metabolism in the liver of PCC-deficient mice.

The PK/PD model described herein will be utilized to inform an optimal mRNA-3927 dose that is expected to yield a substantial reduction of plasma 2-MC in patients with PA. The interspecies predictability of PCCA mRNA between mice, rats, and monkeys based on allometric scaling provided the rationale for allometric prediction of human PK, with the concentration of PCC protein needed for 50% suppression of plasma 2-MC assumed to be similar in mice and humans. Evaluating a wide range of dose levels and regimens to determine an appropriate dose and schedule for novel agents in Phase 1 clinical studies can be very difficult; this is particularly evident for small patient populations, as it can be time consuming, costly, or not feasible, and may be disadvantageous for patients who are exposed to suboptimal or supratherapeutic dose levels.

Translational PK/PD models are increasingly utilized to support clinical development by allowing more efficient patient enrollment and dose selection for rationally designed trials [15,16]. Results from the current model will be used to inform the design of the ongoing first-in-human Phase 1/2 study evaluating mRNA-3927 in participants 1 year of age and older with PA (NCT04159103) [17]. Our study represents further progress toward a disease-modifying treatment that could significantly improve outcomes in patients with PA.

## Conclusions

This work illustrates how a PK/PD model was used to facilitate the quantitative translation of preclinical pharmacology data for an investigational mRNA therapeutic to the clinic in support of selecting an efficacious dose for first-in-human study. The application of such approaches is expected to increase the rate of success in the clinic by ensuring the identification of an optimal efficacious starting dose and rational design of clinical studies. Although the described application is specific to the novel mRNA-3927, the underlying principles and assumptions employed are generally relevant to translational pharmacology efforts for other mRNA therapeutics.

## Acknowledgments

Medical writing assistance was provided by Lara Kallal of MEDiSTRAVA in accordance with Good Publication Practice (GPP3) guidelines, funded by Moderna, Inc., and under the direction of the authors.

## Supplementary Material

Supplemental data

Supplemental data
