# Supplementary material for: Translational Pharmacokinetic/Pharmacodynamic Model for mRNA-3927, an Investigational Therapeutic for the Treatment of Propionic Acidemia
Source: Nucleic Acid Ther. 2023 Mar 30;33(2):141–7. doi: 10.1089/nat.2022.0036 (PMC10066765; doi:10.1089/nat.2022.0036)
Supplement: Supplemental data [file Suppl_TableS1.pdf]

Table S1. PK/PD Model Equations

(A) PCCA/PCCB mRNA PK model

$d(A_1)/dt = input - CL_{12}*C_1$
$d(A_2)/dt = CL_{12} * C_1 - CL_{23} * C_2 + CL_{32} * C_3 - CL_{20} * C_2$
$d(A_3)/dt = CL_{23} * C_2 - CL_{32} * C_3$
$C_1 = A_1/V$<br>$C_2 = A_2/V_2$<br>$C_3 = A_3/V$<br>$C_{13} = C_1 + C_3$
$CL_{12} = tvCL_{12} * (BW/0.025) **cl\alpha$<br>$CL_{32} = tvCL_{32} * (BW/0.025) **cl\alpha$<br>$CL_{23} = tvCL_{23} * (BW/0.025) **cl\beta$<br>$CL_{20} = tvCL_{20} * (BW/0.025) **cl\beta$
$V = tvV * (BW/0.025) **1$<br>$V_2 = tvV_2 * (BW/0.025) **1$

A_1, amount in compartment 1 (plasma compartment); A_2, amount in compartment 2 (tissue compartment); A_3, amount in compartment 3 (plasma compartment); BW, body weight; C_1, concentration in compartment 1; C_2, concentration in compartment 2 (tissue compartment); C_3, concentration in compartment 3; CL_{12}, clearance from compartment 1 to compartment 2; CL_{20}, tissue elimination clearance; CL_{23}, clearance from compartment 2 to compartment 3; CL_{32}, clearance from compartment 3 to compartment 2; V, distribution volume of compartment 1 and 3; V_2, distribution volume of compartment 2; C_{13}, total sum of concentrations (C_1 and C_3) in the plasma compartments; clα, allometric exponent for CL_{12} and CL_{32} parameters (clearance from plasma); clβ, allometric exponent for CL_{23} and CL_{20} parameters (clearance from tissue); mRNA, messenger RNA; PD, pharmacodynamic; PK, pharmacokinetic; tv, typical value.

(B) PCC PD model

$d(C_e)/dt = K_{e0} * (C_{13} - C_e)$
$d(PCC)/dt = K_{syn} * C_e + K_q*(PCP-PCC) - K_{deg} * PCC$
$d(PCP)/dt = K_q*(PCC-PCP)$
$K_{syn} = C_e * Slope$

C_{13}, plasma concentration of mRNA-3927; C_e, effect compartment concentration of mRNA-3927; K_{deg}, PCC protein degradation rate; K_{e0}, equilibrium rate constant for effect compartment; K_q, intercompartmental rate constant for PCC protein; K_{syn}, PCC protein synthesis rate; PCC, propionyl CoA carboxylase; PCP, PCC protein in the peripheral compartment; PD, pharmacodynamic.

(C) 2-MC, 3-HP, C3/C2 PD model

$2\text{-}MC = E0_{2\text{-}MC} + [2\text{-}MC_{base} * (1 - I_{max} * PCC/(IC50_{2\text{-}MC} + PCC))]$
$3\text{-}HP = E0_{3\text{-}HP} + [3\text{-}HP_{base} * (1 - I_{max} * PCC/(IC50_{3\text{-}HP} + PCC))]$
$C3/C2 = E0_{C3/C2} + [C3/C2_{base} * (1 - I_{max} * PCC/(IC50_{C3/C2} + PCC))]$

2-MC, 2-methylcitrate; $2\text{-}MC_{base}$, baseline 2-MC amenable to suppression; 3-HP, 3-hydroxypropionate; $3\text{-}HP_{base}$, baseline 3-HP; C2, acetyl carnitine; C3, propionyl carnitine; $C3/C2_{base}$, baseline C3/C2; $E0_{2\text{-}MC}$, plasma 2-MC levels not affected by mRNA-3927; $E0_{3\text{-}HP}$, plasma 3-HP levels not affected by mRNA-3927; $E0_{C3/C2}$, plasma C3/C2 levels not affected by mRNA-3927; $IC50_{2\text{-}MC}$, PCC concentration needed for 50% of maximal 2-MC reduction; $IC50_{3\text{-}HP}$, PCC concentration needed for 50% of maximal 3-HP reduction; $IC50_{C3/C2}$, PCC concentration needed for 50% of maximal C3/C2 reduction; I_{max}, maximum inhibition; PCC, propionyl CoA carboxylase; PD, pharmacodynamic.
