# Supplementary material for: Translational Pharmacokinetic/Pharmacodynamic Model for mRNA-3927, an Investigational Therapeutic for the Treatment of Propionic Acidemia
Source: Nucleic Acid Ther. 2023 Mar 30;33(2):141–7. doi: 10.1089/nat.2022.0036 (PMC10066765; doi:10.1089/nat.2022.0036)
Supplement: Supplemental data [file Suppl_FigS1-S3.pdf]

Figure S1. Observed and Fitted Individual Concentration–Time Profiles of Plasma 2-MC in Mice

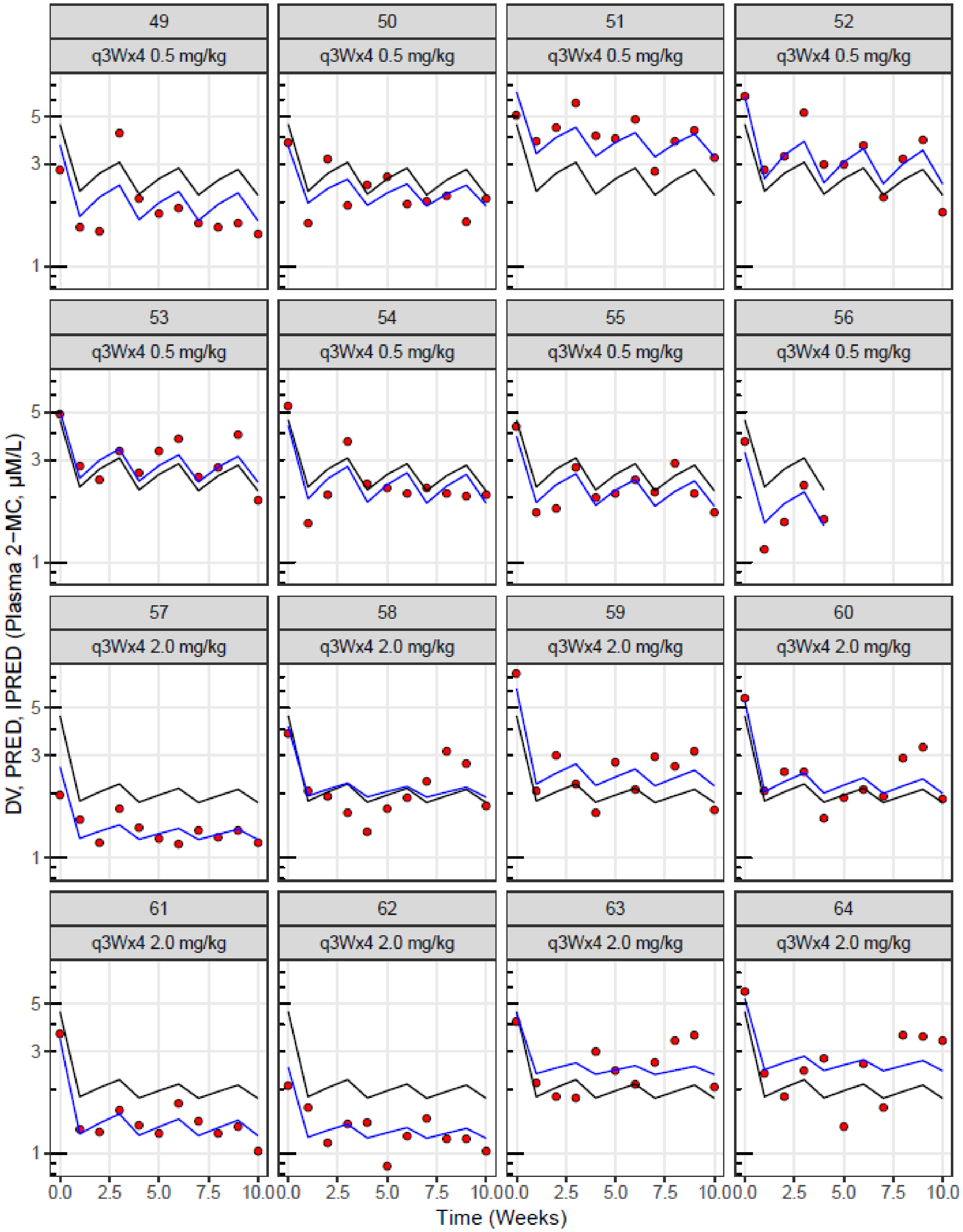

Black lines indicate population predicted, blue lines indicate individual predicted, and closed circles indicate observations. 2-MC, 2-methylcitrate; DV, dependent variable; IPRED, individual predicted; PRED, population predicted; q3W, every 3 weeks.

Figure S2. Observed and Fitted Individual Concentration–Time Profiles of Plasma 3-HP in Mice

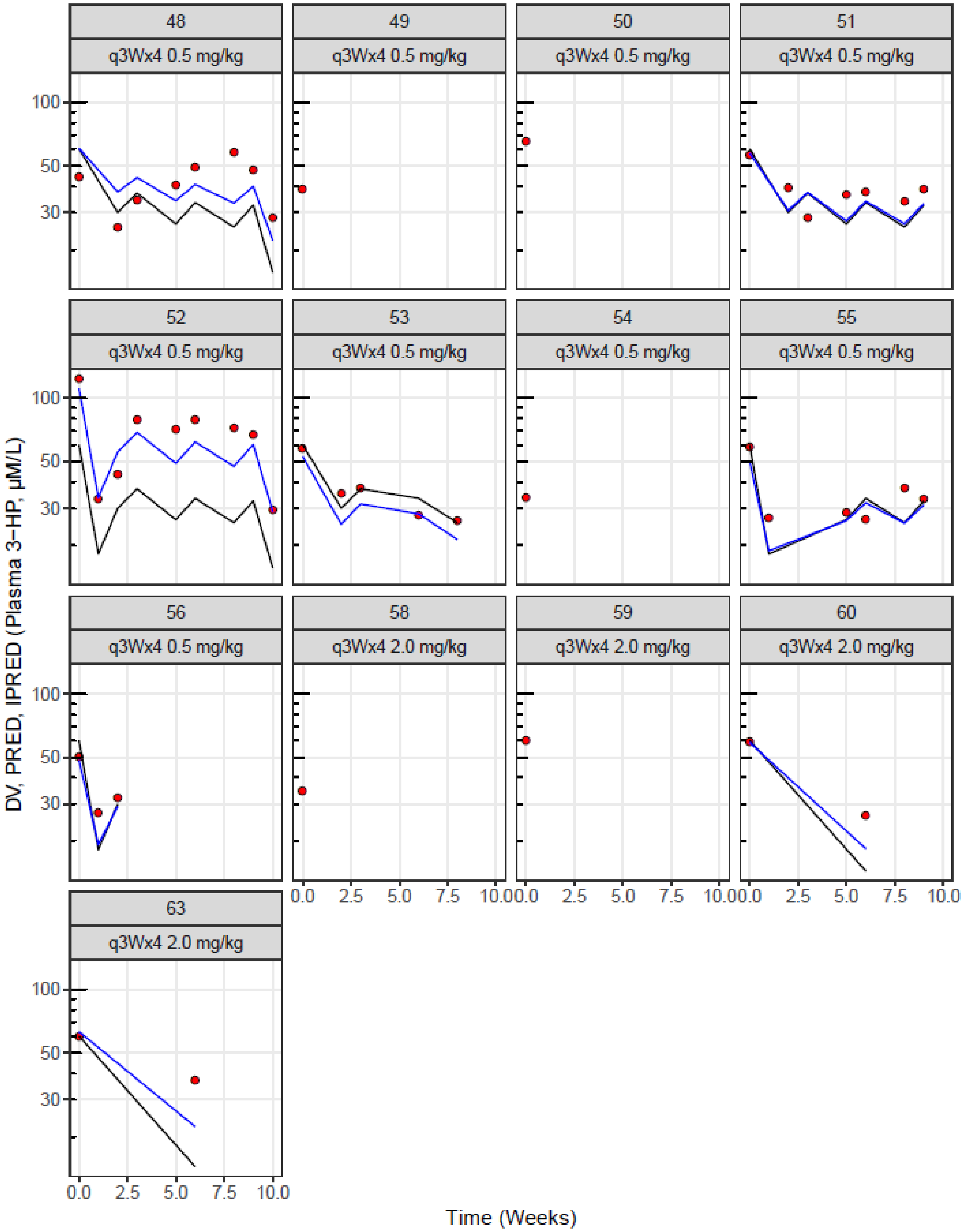

Black lines indicate population predicted, blue lines indicate individual predicted, and closed circles indicate observations. 3-HP, 3-hydroxypropionate; DV, dependent variable; IPRED, individual predicted; PRED, population predicted; q3W, every 3 weeks.

Figure S3. Observed and Fitted Concentration–Time Profiles of Plasma C3/C2 Ratio in Mice

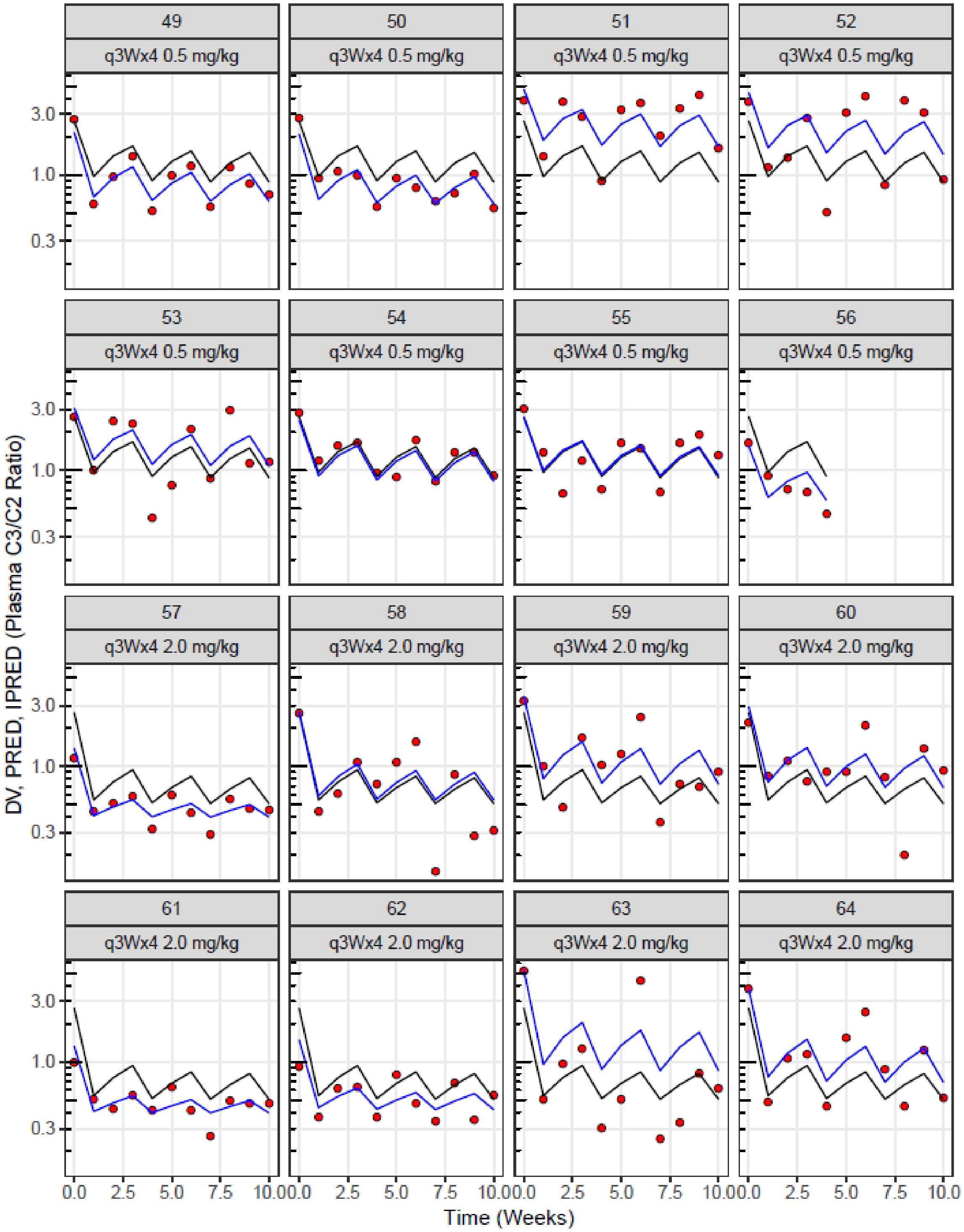

Black lines indicate population predicted, blue lines indicate individual predicted, and closed circles indicate observations. C2, acetyl carnitine; C3, propionyl carnitine; DV, dependent variable; IPRED, individual predicted; PRED, population predicted; q3W, every 3 weeks.
